# EGCG induces human mesothelioma cell death by inducing reactive oxygen species and autophagy

**DOI:** 10.1186/1475-2867-13-19

**Published:** 2013-02-23

**Authors:** Motohiko Satoh, Yukitoshi Takemura, Hironobu Hamada, Yoshitaka Sekido, Shunichiro Kubota

**Affiliations:** 1 Department of Life Sciences, Graduate School of Arts and Sciences, The University of Tokyo, 3-8-1 Komaba, Meguro-ku, Tokyo, 153-8902, Japan; 2 Graduate School of Biomedical and Health Sciences, Hiroshima University, Hiroshima, 734-8553, Japan; 3 Division of Molecular Oncology, Aichi Cancer Center Research Institute, Nagoya, Aichi, 464-8681, Japan; 4 Department of Cancer Genetics, Nagoya University Graduate School of Medicine 65, Nagoya, Aichi, 466-8550, Japan

**Keywords:** Mesothelioma, EGCG, Reactive oxygen species, Apoptosis, Autophagy, Chloroquine

## Abstract

Malignant mesothelioma is an asbestos-related fatal disease with no effective cure. We studied whether a green tea polyphenol, epigallocathechin-3-gallate (EGCG), could induce cell death in five human mesothelioma cell lines. We found that EGCG induced apoptosis in all five mesothelioma cell lines in a dose-dependent manner. We further clarified the cell killing mechanism. EGCG induced reactive oxygen species (ROS), and impaired the mitochondrial membrane potential. As treatment with ROS scavengers, catalase and tempol, significantly inhibited the EGCG-induced apoptosis, ROS is considered to be responsible for the EGCG-induced apoptosis. Further, we found that EGCG induced autophagy, and that when autophagy was suppressed by chloroquine, the EGCG-induced cell death was enhanced. Taken together, these results suggest that EGCG has a great potential for the treatment of mesothelioma by inducing apoptosis and autophagy.

## Background

Malignant mesothelioma is an aggressive tumor associated with asbestos exposure. The worldwide incidence of mesothelioma is expected to increase [1,2]. Although many clinical treatments including surgery, radiotherapy and chemotherapy have been reported, the prognosis of patients remains poor [3,4].

We recently reported that treatment with a high dose of ascorbic acid brought about the death of human mesothelioma cells by inducing reactive oxygen species (ROS), which leads to oxidative stress, and subsequently, to cell death [5]. We hypothesized that epigallocatechin-3-gallate (EGCG) may induce mesothelioma cell death by inducing reactive oxygen species, because EGCG is known to be involved in oxidative stress [6,7]. The effects of EGCG on mesothelioma growth have not yet been sufficiently studied. Only two papers reported by the same group are available concerning the effects of EGCG on mesothelioma cell death [8,9]. In one paper Burlando et al. reported that EGCG induced cell death via H_2_O_2_-dependent T-type Ca^2+^ channel opening [8]. Their data are not inconsistent with our hypothesis that EGCG may induce mesothelioma cell death via oxidative stress. Although we had no data concerning H_2_O_2_-dependent T-type Ca^2+^ channel opening, we tested our idea that EGCG might induce autophagy in the present study.

The main source of ROS is in the mitochondria, which play pivotal roles in cell survival and cell death such as apoptosis and autophagy. Autophagy is a lysosomal degradation process involved in a wide range of physiological and pathological processes that is often induced under conditions of oxidative stress that could lead to cell death [10-13]. Autophagy has been implicated in many diseases, including cancer [12], where it apparently has dual roles, acting as both a tumor suppressor and as a tumor survival or growth factor. Several reports have suggested that inhibition of autophagy restores chemosensitivity and augmentes tumor cell death [13-19]. The inhibition of autophagy can be achieved by using chloroquine (CQ) in combination with chemotherapy or targeted agents [20-22].

In the present study, we demonstrate that EGCG induced the death of five human mesothelioma cell lines. We further showed that the mechanism of the cell death occurred via ROS production and a reduction in the mitochondrial membrane potential. Moreover, we found that EGCG induced autophagy, and that the inhibition of autophagy by CQ enhanced the EGCG-induced cell death.

## Results

### EGCG inhibits mesothelioma cell growth

**Figure 1:**
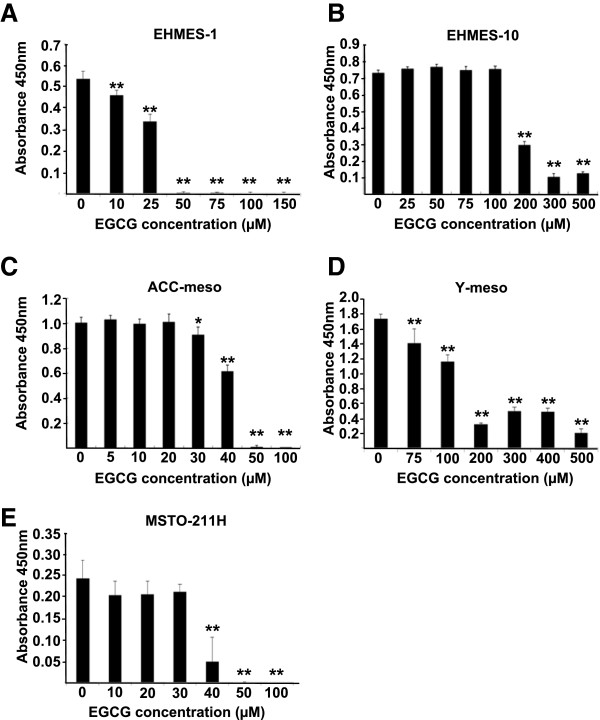
**Effect of EGCG on mesothelioma cell growth.** The mesothelioma cells were treated with EGCG (10 μM to 500 μM) for 24 h. The viability of the cells was evaluated with cell counting kit assay. Values are expressed as absorbance at 450 nm. The results are expressed as Mean ± S.D. (n=6). **P < 0.01, *P < 0.05.

We first investigated whether EGCG inhibited cell growth using five human mesothelioma cell lines (EHMES-10, EHMES-1, ACC-meso, Y-meso and MSTO-211H). The mesothelioma cells were treated with EGCG for 24 h at concentrations from 10 μM to 500 μM. EGCG reduced the cell viability in a dose-dependent manner (Figure 1). Differences in the sensitivity to EGCG were observed among the five cell lines. The concentrations required to inhibit the growth of the cells by 50% (IC_50_) values were 179, 29, 42, 128 and 35 μM for the EHMES-10, EHMES-1, ACC-meso, Y-meso, and MSTO-211H cells, respectively.

### Signal transduction induced by EGCG

**Figure 2:**
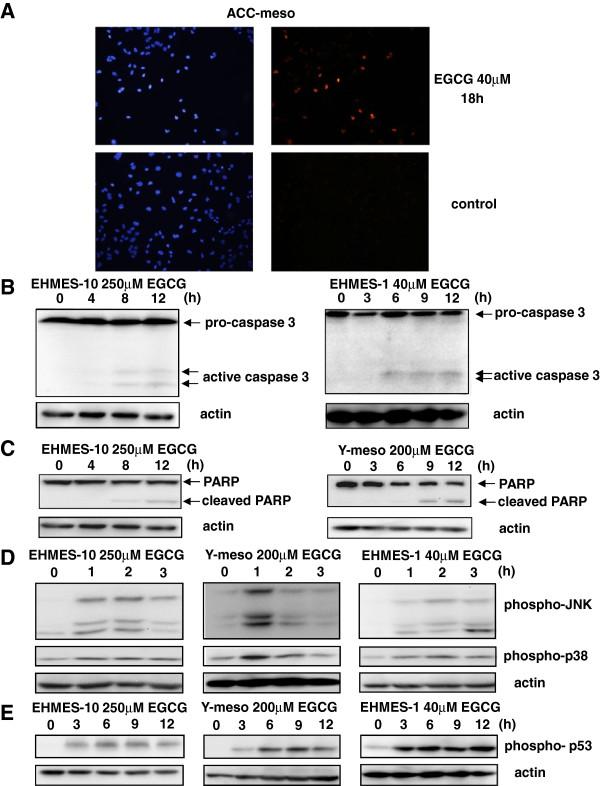
**Effects of EGCG on apoptotic signaling pathway in mesothelioma cells.** (**A**) TUNEL assay was performed (right panel). The left panel shows DAPI staining. (**B**) Active fragments of casapase-3 were analyzed. (**C**) Cleaved PARP fragments were analyzed. (**D**) Expression levels of phospho-JNK and phospho-p38 were analyzed. (**E**) Phospho-p53 expression levels were analyzed.

We next studied the signal transduction involved in EGCG-induced mesothelioma cell growth inhibition. We studied apoptosis-related signal transduction to elucidate whether the EGCG-induced mesothelioma cell growth inhibition was due to apoptosis. Using the TUNEL assay we found that EGCG induced mesothelioma cell apoptosis in EHMES-10, EHMES-1, ACC-meso, and Y-meso cells. As similar results (many TUNEL positive cells) were obtained for all four of these cell lines, only the data for the ACC-meso cells are shown in Figure 2A.

As caspase-3 activation and PARP cleavage are hallmark of apoptosis, we next studied caspase-3 activation and PARP cleavage using a Western blot analysis. As expected, EGCG induced caspase-3 fragments in both EHMES-10 and EHMES-1cells (Figure 2B), and PARP cleavage in both EHMES-10 and Y-meso cells (Figure 2C). As phospho-JNK, phospho-p38 and phospho-p53 also play pivotal roles in apoptotic signaling, we next studied whether EGCG induced expression of phospho-JNK, phospho-p38 and phospho-p53 using western blotting. EGCG increased the expression levels of phospho-JNK, phospho-p38 and phospho-p53 in EHMES-10, Y-meso and ACC-meso cells after 1–3 hr (Figure 2D and 2E). All these data about signal transduction are consistent with the idea that EGCG induces apoptosis in mesothelioma cells.

### Mesothelioma cells produce reactive oxygen species (ROS) following EGCG treatment

**Figure 3:**
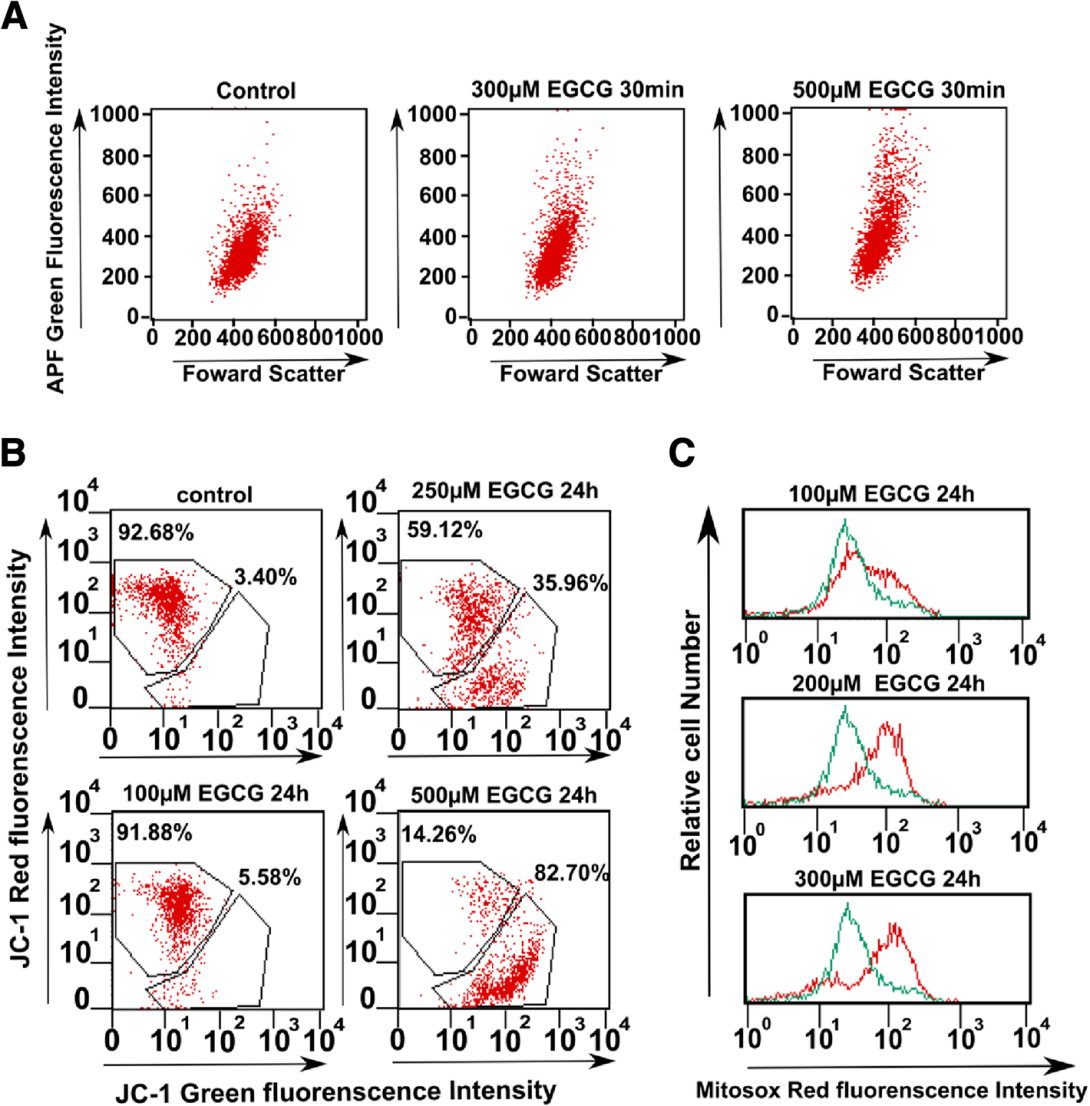
**Effect of EGCG on intracellular and mitochondrial ROS production, and mitochondrial membrane potential in mesothelioma cells.** (**A**) Intracellular superoxide was analyzed using APF. (**B**) JC-1 fluorescence was analyzed. (**C**) Mitochondrial superoxide was analyzed by MitoSOX.

We next studied the mechanism responsible for the apoptosis induced by EGCG using EHMES-10 cells. Since EGCG has been demonstrated to be involved in oxidative stress [6,7], we first analyzed the ROS production inside the cells. To determine whether EGCG leads to ROS production, experiment was performed using APF (a ROS probe), which could detect ROS such as •OH, ONOO- and OCl-. After the treatment with 500 μM of EGCG for 30 min, the fluorescence intensity was examined in EGCG-treated cells (Figure 3A). The results suggest that EGCG induced ROS production inside the cells. Therefore, we focused on mitochondria where ROS are produced. The EHMES-10 cells were treated with EGCG (100, 250 and 500 μM) for 24 hr, and were incubated with 2 μg/ml JC-1 for 30 min. The JC-1 fluorescence was analyzed by FACS. The number of cells with conventional mitochondrial membrane potential (JC-1 red fluorescence) was decreased (91.88%, 59.12% and 14.26%, respectively), while that of cells with a low mitochondrial membrane potential (JC-1 green fluorescence) was increased in a dose-dependent manner (5.58%, 35.96% and 82.7%, respectively) (Figure 3B). These results suggest that EGCG decreased mitochondrial membrane potential.

We next examined the production of superoxide in mitochondria by a FACS analysis, using MitoSOX Red, a mitochondrial superoxide indicator. EHMES-10 cells were treated with EGCG concentrations of 100, 200 and 300 μM for 24 hr, and then, the cells were incubated with MitoSOX Red for 15 min. The mean fluorescence intensity of MitoSOX Red was increased in a dose-dependent manner (Figure 3C). These data suggest that EGCG led to the production of superoxide in the mitochondria of EHMES-10 cells.

### Catalase and tempol inhibit EGCG-induced cell death

**Figure 4:**
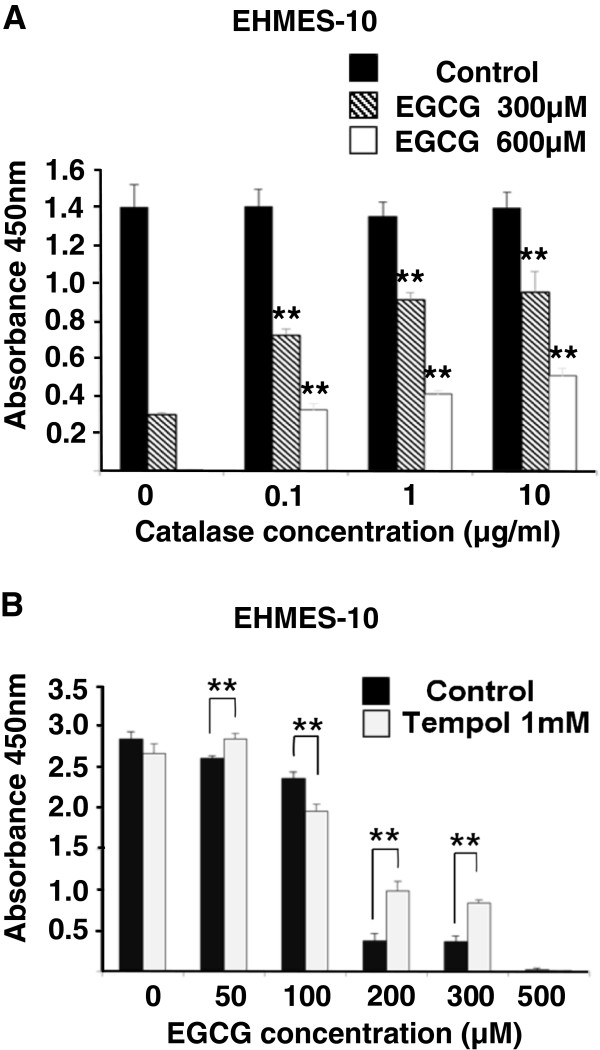
**Catalase and tempol restore the cell death induced by EGCG.** EHMES-10 cells were incubated with EGCG together with catalase (**A**) or together with tempol (**B**). The viability was evaluated using cell counting kit assay. Values are expressed as absorbance at 450 nm. The results are expressed as Mean ± S.D. (n=6). **P < 0.01.

We next studied whether the ROS production was responsible for the EGCG-induced EHMES-10 cell death using the H_2_O_2_-scavenger catalase and superoxide-scavenger tempol [23-26]. EHMES-10 cells were incubated with EGCG alone, EGCG+catalase, or EGCG+tempol and then, the viability of the cells was analyzed using a cell counting kit. Catalase (0.1, 1 and 10 μg/ml) significantly prevented the EGCG-induced cell death (Figure 4A). Tempol (1 mM) also significantly inhibited the cell death induced by 200 μM EGCG or 300 μM EGCG (Figure 4B), but not that induced by 50 μM or 100 μM EGCG. These data suggest that H_2_O_2_ and superoxide are responsible for the EGCG-induced EHMES-10 cell death.

### EGCG induces autophagy, and the inhibition of autophagy enhances EGCG-induced cell death

**Figure 5:**
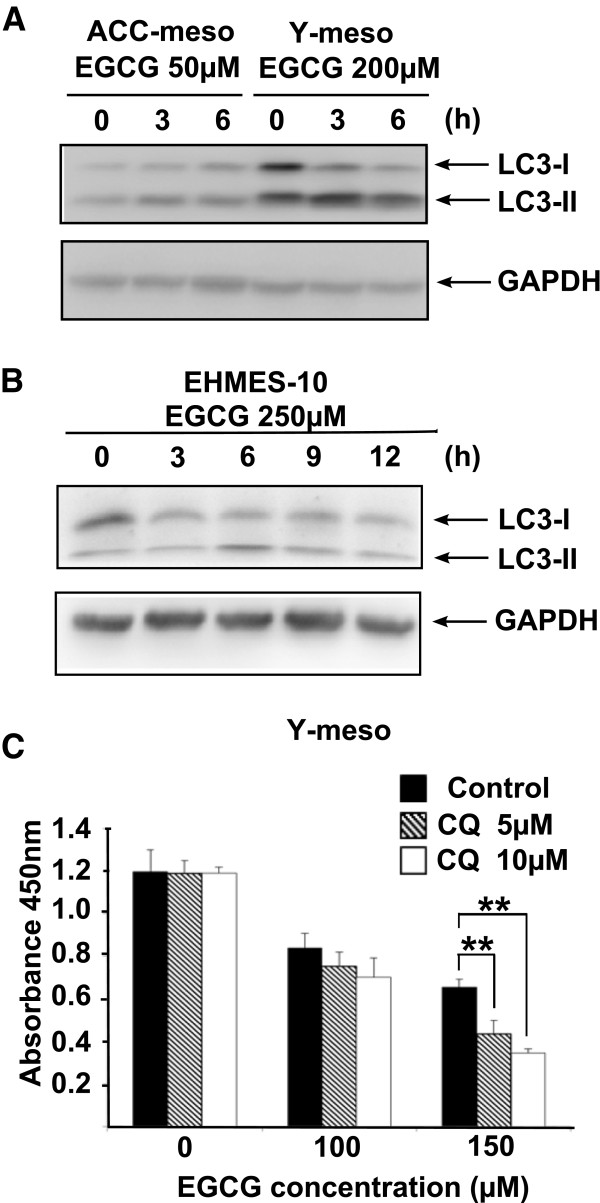
**Effect of EGCG on autophagy and the effect of CQ on cell death.** (**A** and **B**) Western blot analysis was performed using LC-3 specific antibody or GAPDH antibody. (**C**) Y-meso cells were incubated with EGCG together with CQ. Cell viability was analyzed using cell counting kit. Values are expressed as absorbance at 450 nm. The results are expressed as Mean ± S.D. (n=6). **P < 0.01.

As both apoptosis and autophagy are triggered by common upstream signals [27,28], we examined whether EGCG induces autophagy using ACC-meso, Y-meso and EHMES-10 cells. The LC3-II expression levels were examined using a specific antibody against LC3 in a Western blot analysis. ACC-meso, Y-meso and EHMES-10 cells were treated with EGCG (50, 250 and 200 μM, respectively) for 3, 6, 9 and 12hr. Then, the expression levels of LC3-I and LC3-II were analyzed. EGCG increased the LC3-II expression levels at 3 and 6 hr after treatment (Figure 5A and 5B). These data suggest that EGCG induced autophagy in mesothelioma cells.

We further examined whether the inhibition of autophagy affected the EGCG-induced cell death in Y-meso cells. Y-meso cells were treated with chloroquine (CQ)(5 and 10 μM), an autophagy inhibitor and with EGCG (100 and 150 μM) for 24 hr. Next, the cell viability was analyzed using a cell counting kit. The addition of chloroquine (CQ) at concentrations of 5 and 10μM enhanced the EGCG (150 μM)-induced cell death in a dose-dependent manner, but CQ did not affect the cell death induced by 100 μM EGCG (Figure 5C). These data indicate that the inhibition of autophagy enhances EGCG-induced cell death.

## Discussion

In this study, we demonstrated that EGCG induced human mesothelioma cell death in a dose-dependent manner. We further clarified the mechanism responsible for such cell killing. EGCG induced reactive oxygen species (ROS) and impaired the mitochondrial membrane potential. The use of ROS scavengers, catalase and tempol, significantly inhibited the EGCG-induced apoptosis. Furthermore, we found that EGCG induced autophagy, and that the suppression of autophagy enhanced the EGCG-induced cell death.

There are many reports about the effects of EGCG on cancer cell growth [7,29,30]. However, there are only two papers concerning EGCG-induced mesothelioma cell death [8,9]. In the former paper it was reported that the EGCG-induced cell death occurred via H_2_O_2_-dependent T-type Ca^2+^ channel opening. The data are not inconsistent with our present data showing that EGCG-induced mesothelioma cell death occurs via the production of ROS (H_2_O_2_ and superoxide). As we did not analyze the H_2_O_2_-dependent T-type Ca^2+^ channel opening, it is unclear whether H_2_O_2_-dependent T-type Ca^2+^ channel opening is involved in our case.

As both apoptosis and autophagy are triggered by common upstream signals [27,28], we tested whether EGCG induced autophagy, and found that it did induce autophagy, and that treatment of cells with CQ, an autophagy inhibitor, augmented the EGCG-induced cell death. Autophagy is known to play dual roles in cancer, acting as both a tumor inhibitor and as a tumor growth promoter [12]. In our present study, autophagy protected the mesothelioma cells from death. These data are consistent with several reports in other cancer cells demonstrated that the inhibition of autophagy restored chemosensitivity and augmented tumor cell death [13-19]. CQ is a well-known drug that is widely used for the prophylaxis treatment of malaria because of both its efficacy and low toxicity to humans [31-33]. It is also widely used as an anti-rheumatoid agent [31], and our data suggests that it may be useful for treating mesothelioma patients if used in combination with EGCG.

The cell death induced by EGCG was prevented by treatment with catalase, thus suggesting that the effects of EGCG were largely due to the production of hydrogen peroxide by the cells. Because the catalase was added extracellularly, it could decrease the hydrogen peroxide that was extracellularly induced by EGCG. In contrast, tempol, a membrane-permeable radical scavenger, also prevented the EGCG-induced cell death. This reagent reduced the formation of the hydroxyl radical by scavenging superoxide anions. These results suggest that the superoxide anion produced in the cells could lead to cell death either directly or indirectly. Therefore, EGCG treatment may induce the disruption of the mitochondrial membrane potential inside cells. In fact, as shown in Figure 3B, EGCG did decrease the mitochondrial membrane potential.

Several studies report that EGCG has dual function of anti-oxidant and pro-oxidant potential [34,35]. Low concentrations (i.e. 10 μM) of EGCG scavenged free radicals thereby inhibiting oxidative damage to cellular DNA. In contrast, higher concentrations (i.e. 100 μM) of EGCG induced cellular DNA damage [34]. Dual function of EGCG in normal human lymphocytes is reported in [35]. In our present study we have shown similar results as shown in Figure 1. In most mesothelioma cell lines higher concentrations (i.e. 100 μM or 200 μM) of EGCG induced cell death and low concentrations (i.e. 10 μM) of EGCG failed to induce cell death.

The accumulating experimental evidence that cancer cells are more susceptible to hydrogen peroxide and to hydrogen peroxide-induced cell death than normal cells was discussed in a mini-review paper [36]. However, it is unclear what specific concentrations of hydrogen peroxide are required to kill cancer cells. It has been speculated that hydrogen peroxide may be present at low levels in normal cells because there are higher levels of catalase activity.

## Conclusion

We have herein shown that EGCG induced apoptosis in five human mesothelioma cell lines. We further demonstrated that the mechanism responsible for the EGCG-induced cell death was via ROS production and a decrease in the mitochondrial membrane potential. Moreover, we found that EGCG induced autophagy, and that the inhibition of autophagy by CQ enhanced the EGCG-induced cell death. These data suggest that EGCG may be useful for the treatment of malignant mesothelioma. *In vivo* animal experiments using EGCG in combination with CQ are currently underway in our laboratory to confirm these effects and as a first step toward the clinical application of this treatment.

## Materials and methods

### Cell culture and reagents

Five mesothelioma cell lines, ACC-meso 1 (ACC-meso) [37], Y-meso 8A (Y-meso [37], EHMES-10 [38,39], EHMES-1 [38], and MSTO-211H (purchased from ATCC, Manassas, VA) were used in this study. ACC-meso and Y-meso were cultured in DMEM (Dulbecco’s modified Eagle’s medium) (Sigma, St. Louis, MO) supplemented with 10% fetal calf serum and 1× penicillin–streptomycin antibiotics (Wako Pure Chemical Industries Ltd., Osaka, Japan). EHMES-10, EHMES-1 and MSTO-211H were cultured in RPMI-1640 (Sigma) supplemented with 10% fetal calf serum and 1×penicillin–streptomycin antibiotics. All cell lines were incubated at 37°C in 5% CO_2_.

### Cell viability assay

Cells were seeded at a density of 2,000 cells/well in 96-well plate and treated with EGCG at various concentrations for 24 h. To assess the activity in the presence of anti-oxidative agents, cells were treated with EGCG (Sigma-Aldrich, Tokyo, Japan) or EGCG with tempol (4-hydroxy-2,2,6,6-tetramethylpiperidine-N-oxyl) (Sigma-Aldrich) or with EGCG and catalase (Sigma-Aldrich) for 24 h. The cell viability was determined using the Cell Counting Kit-8 (Dojindo Laboratories, Kumamoto, Japan). The color intensity was measured in a microplate reader (Thermo Electron Corporation, Vantaa, Finland) at 450 nm.

### Western blotting analysis

After EGCG treatment, cells were lysed in Triton X-100 lysis buffer (1% Triton X-100, 10% glycerol, 150 mM NaCl, 2 mM EDTA, 0.02% NaN_3_, 10 μg/ml PMSF, and 1 mM Na_3_VO_4_). Total cell lysates were separated by SDS-PAGE and transferred to polyvinylidene difluoride (PVDF) membranes. The membranes were reacted with a rabbit anti-PARP (Poly ADP-ribose polymerase) antibody, anti-phosho-p53 (ser20) antibody, anti-phospho JNK (c-Jun N-terminal protein kinase) antibody, anti-phosho-p38 antibody, anti-actin antibody, and anti-caspase-3 antibody (New England Biolabs, Ipswich, MA) followed by a peroxidase-conjugated anti-rabbit IgG antibody (New England Biolabs). In other experiments, membranes were reacted with a mouse anti-LC3 (microtubule associated protein 1 light chain-3) monoclonal antibody (nanoTools, Teninge, Germany), and an anti-GAPDH monoclonal antibody (Santa Cruz Biotechnology, Inc., Santa Cruz, CA) followed by a peroxidase-conjugated anti-mouse IgG antibody (New England Biolabs). Proteins were then visualized using Immobilon Western reagents (Millipore, Billerica, MA).

### Mitochondrial membrane potential and superoxide detection

J-aggregate-forming lipophilic cation (JC-1) (Wako Pure Chemical Industries Ltd.) was used to evaluate the mitochondrial membrane potential. For these experiments, EHMES-10 cells were seeded on 24-well plate. After EGCG treatment for 24 h, the cells were washed with PBS containing 10% fetal calf serum (10% FCS-PBS) and incubated with 2 μg/ml JC-1 (final concentration) in 10% FCS-PBS for 30 min at 37°C.

Intracellular superoxide was detected using 3^′^-p-(aminophenyl) fluorescein(APF)(SEKISUI MEDICAL CO. LTD., Tokyo, Japan). EHMES-10 cells were seeded on 12-well plate, and the cells were incubated with 5 μM APF (final concentration) for 30 min at 37°C. After washing with medium, the cells were treated with EGCG for 30min. Then, the cells were re-suspended in 500 μl of warm culture medium and analyzed by a FACS Calibur instrument (BD, Franklin Lakes, NJ).

Mitochondrial superoxide in living cells was detected using MitoSOX (Invitrogen, Eugene, OR). EHMES-10 cells were incubated for 24 h. Then, the cells were incubated with MitoSOX Red (final concentration 5 μg/ml) for 15 min at 37°C. After being washed with warm culture medium, the cells were re-suspended in 500 μl of warm culture medium and analyzed by a FACS Calibur instrument.

### TUNEL assay

ACC-meso cells were seeded on LabTek chamber slides (Nalge Nunc International, Rochester, NY) and incubated with 100 μM EGCG for 16 h at 37°C. Then, the cells were washed twice with PBS (phosphate–buffered saline) and fixed with 3% formaldehyde in PBS for 30 min. The fixed cells were stained using the terminal dUTP nick-end labeling (TUNEL) method, using an *In Situ* Cell Death Detection Kit, TMR (Roche Applied Science, Mannheim, Germany), according to the manufacturer’s instructions.

### Statistical analysis

The results are expressed as the means±standard deviation. The means were compared to those of untreated control cells using Student’s t-test. One way ANOVA with a Bonferroni multiple comparison post-hoc test was performed when more than two groups were compared using the Excel Statcel 3 software program (purchased from the publisher OMS Ltd., Tokyo, Japan). A probability value<0.05 was considered to be statistically significant.

## Abbreviations

EGCG: Epigallocathechin-3-gallate; ROS: Reactive oxygen species; CQ: Chloroquine; MAPK: Mitogen-activated protein kinase; APF: 3^′^-p-(aminophenyl) fluorescein; JC-1: J-aggregate-forming lipophilic cation; TUNEL: Terminal dUTP nick-end labeling.

## Competing interests

The authors declare that we have no conflict of interest.

## Authors’ contribution

MS and YT performed experiments and collected data. HH established two cell lines, EHMES-1 and EHMES-10. YS established two cell lines, ACC-meso and Y-meso. SK designed this study and drafted the article. All authors read and approved the final manuscript.
